# Effects of Varying Spiral-Ring Pitches on CO_2_ Absorption by Amine Solution in Concentric Circular Membrane Contactors

**DOI:** 10.3390/membranes14070147

**Published:** 2024-06-27

**Authors:** Chii-Dong Ho, Jui-Wei Ke, Jun-Wei Lim

**Affiliations:** 1 Department of Chemical and Materials Engineering, Tamkang University, Tamsui, New Taipei 251301, Taiwan; 611400671@o365.tku.edu.tw; 2 HICoE-Centre for Biofuel and Biochemical Research, Department of Fundamental and Applied Sciences, Institute of Self-Sustainable Building, Universiti Teknologi PETRONAS, Seri Iskandar 32610, Malaysia; junwei.lim@utp.edu.my; 3 Department of Biotechnology, Saveetha School of Engineering, Saveetha Institute of Medical and Technical Sciences, Saveetha University, Chennai 602105, India

**Keywords:** spiral ring pitch, CO_2_ absorption, Sherwood number, concentric membrane contactor, concentration polarization

## Abstract

The CO_2_ absorption flux while using monoethanolamide (MEA) solution in a spiral-wired channel was significantly enhanced by optimizing both the descending and ascending spiral ring pitch configurations within the filled channel. In this study, two distinct spiral ring pitch configurations were integrated into concentric circular membrane contactors to augment CO_2_ absorption flux. Spiral rods were strategically inserted to mitigate concentration polarization effects, thereby reducing mass transfer boundary layers and increasing turbulence intensity. A theoretical one-dimensional model was developed to predict absorption flux and concentration distributions across varying MEA absorbent flow rates, CO_2_ feed flow rates, and inlet CO_2_ concentrations in the gas feed. Theoretical predictions of absorption flux improvement were validated against experimental results, demonstrating favorable agreement for both ascending and descending spiral ring pitch operations. Interestingly, the results indicated that descending spiral ring pitch operations achieved higher turbulent intensity compared to ascending configurations, thereby alleviating concentration polarization resistance and enhancing CO_2_ absorption flux with reduced polarization effects. Specifically, under conditions of a 40% inlet CO_2_ concentration and 5 cm^3^/s MEA feed flow rate, a notable 83.69% enhancement in absorption flux was achieved compared to using an empty channel configuration. Moreover, a generalized expression for the Sherwood number was derived to predict the mass transfer coefficient for CO_2_ absorption in concentric circular membrane contactors, providing a practical tool for performance estimation. The economic feasibility of the spiral-wired module was also assessed by evaluating both absorption flux improvement and incremental power consumption. Overall, these findings underscore the effectiveness of optimizing spiral ring pitch configurations in enhancing CO_2_ absorption flux, offering insights into improving the efficiency and economic viability of CO_2_ capture technologies.

## 1. Introduction

Biogas undergoes processing and conditioning to remove impurities such as CO_2_ (30–45%) and H_2_S (0.5–1%), enhancing its utility and meeting pipeline transport requirements. Addressing environmental concerns and global warming issues [1], this process mitigates CO_2_ emissions from fossil fuel combustion flue gases, which are significant greenhouse gases. Membrane contactors, recognized for their high separation efficiency [2], play a crucial role in CO_2_ capture [3] from these emissions [4], leveraging chemical potential differences across hydrophobic membranes. Purification technology is employed to selectively absorb soluble gas mixture components on the membrane surface within liquid/liquid and gas/liquid systems [5,6]. Gas/liquid membrane contactor technology facilitates efficient contact between liquid absorbents and flue gases, offering advantages such as an increased effective contact area, continuous operation, and reduced dimensions of absorption columns [7]. The efficiency of membrane absorption depends heavily on the properties of selective membrane materials [8], including hybrid silica aerogel, highly porous polyvinylidene fluoride (PVDF)/siloxane nanofibrous membranes [9,10], and composite membranes [11], tailored for CO_2_ absorption. Microporous hydrophobic membranes, with relatively low production costs, play a crucial role in selectively separating specific substances, utilizing non-wetted membrane pores at the gas/liquid interface [12], which depends on the distribution coefficient and a composition gradient of gas solute in the gas/liquid system [13]. The dusty gas model [14] is widely employed to describe mass transfer characteristics across membranes, aiding in estimating process performance [15] involving reactions and diffusion [16]. Enhanced CO_2_ capture efficiency is achieved through physical absorption based on CO_2_ solubility in solvents, thereby reducing regeneration costs [17], with alkanolamine solvents [18] commonly utilized due to the associated chemical reactions [19]. Previous research has extensively studied the mechanisms of CO_2_ absorption in MEA solutions [20], highlighting membrane contactors’ potential for alkanolamine-based CO_2_ capture processes.

Previous studies have conducted comprehensive experiments on gas/liquid absorption using shell and tube membrane contactors, employing computational fluid dynamics (CFD) [21]. These experiments have considered laminar liquid flow profiles [22] and aimed to achieve highly efficient CO_2_ removal under turbulent flow conditions. However, the membrane separation process is prone to concentration polarization near the membrane surface, which leads to decreased mass transfer rates. This effect is commonly observed in various membrane separation processes, resulting in reduced trans-membrane mass flux [23,24,25]. To mitigate concentration polarization, turbulence promoters have been implemented to improve flow channels, such as roughened surfaces [26], spacer-filled channels [27], and carbon-fiber spacers [28]. These enhancements effectively increase turbulent intensity within the gas/liquid membrane contactor system, thereby enhancing convective mass transfer coefficients [29] under diverse operational conditions. Inserting a wire spiral into a concentric circular tube effectively modifies the convection flow dynamics, creating secondary flows or eddies that reduce mass transfer resistances across concentration boundary layers [30]. Consequently, this design modification significantly boosts CO_2_ absorption efficiency compared to modules lacking spiral rings. Studies on various spiral ring pitches [31] have highlighted their hydrodynamic influence on mass transfer mechanisms, revealing their capability to overcome increased mass transfer resistance and reduce power consumption. This contrasts with the approach of using a constant spiral ring pitch, which has been previously applied in heavy water enrichment [32] and demonstrated improved device performance with smaller spiral ring-filled channels in earlier work [33]. A forward-looking strategy aims to reduce overall mass transfer resistance by disrupting mass transfer boundary layers and mitigating concentration polarization through dynamic flow adjustments and enhanced turbulent intensity achieved by embedding various spiral ring pitches [34,35]. A concentric circular membrane contactor was designed with a tightly fitted spiral ring in a narrow annular space to enhance CO_2_ absorption flux by minimizing concentration polarization resistance. Previous research has shown that concentration polarization can be mitigated in ring–rod tubular membrane contactors, leading to increased turbulence intensity. However, the isothermal diffusion–reaction process and chemical reactions during MEA absorbent flow can increase mass transfer resistance towards the latter half of the module, exacerbating concentration polarization. In this study, device performance was further optimized by incorporating various spiral wire pitches along the flow channel. This approach aims to adjust the hydraulic diameter and spiral ring pitches to achieve a specified volumetric feed rate, effectively reducing undesired resistance while ensuring a manageable increase in power consumption for economic viability.

In this study, device performance was enhanced by integrating descending and ascending spiral ring pitches along the flow channel. The theoretical development included mass balance considerations and chemical reaction analyses, complemented by experimental validation using a spiral-ring concentric circular module made of PTFE/PP (polytetrafluoroethylene/polypropylene). The effectiveness of the absorption process was evaluated through a mass transfer enhancement factor, assessing the impacts of spiral wire pitch, CO_2_ feed concentration, and MEA feed flow rate on CO_2_ absorption flux under varying spiral ring pitch configurations. The mass transfer behaviors across the membrane exhibited distinct characteristics, and mathematical formulations were developed building upon prior studies [33], successfully improving membrane absorption rates. Additionally, the incorporation of turbulence promoters in membrane contactor systems was found to increase pressure drop in the feed channel [36]. Thus, the study also evaluated the trade-off between achieving high CO_2_ absorption flux and managing energy consumption, aiming to provide an economic assessment for membrane module applications [37]. Gas absorption mechanisms within the spiral ring-filled module were investigated through modeling mass balance equations and chemical reactions using PTFE membranes. This approach facilitated a balanced consideration between enhancing CO_2_ absorption flux and managing energy consumption, ensuring technical and economic feasibility in designing gas membrane absorption modules. The mathematical formulation developed for spiral-ring concentric circular tubes in this study represents a valuable contribution, potentially applicable to various hydrophobic membrane systems.

## 2. Theory and Analysis

**Figure 1:**
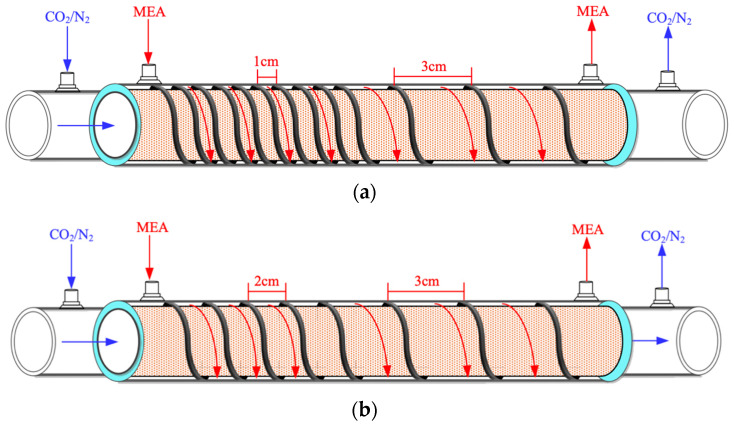

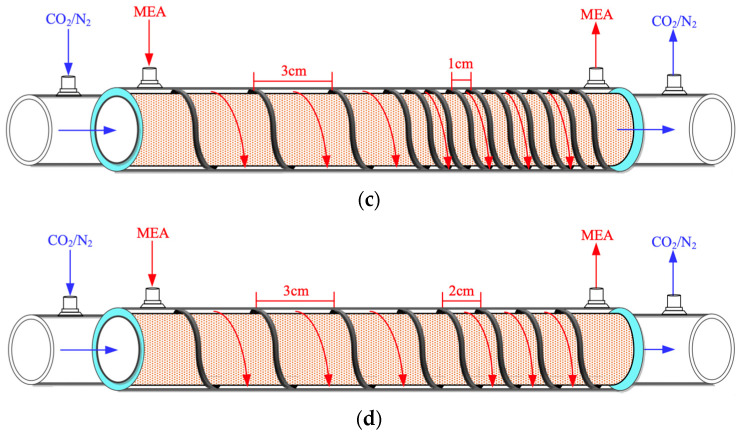
Spiral-ring concentric circular membrane contactors. (**a**) Membrane contactor with ascending (1 cm to 3 cm) spiral ring pitch operations. (**b**) Membrane contactor with ascending (2 cm to 3 cm) spiral ring pitch operations. (**c**) Membrane contactor with descending (3 cm to 1 cm) spiral ring pitch operations. (**d**) Membrane contactor with descending (3 cm to 2 cm) spiral ring pitch operations.

This study presents mathematical modeling of CO_2_ absorption in an MEA solution flowing through the shell side of a spiral-ring concentric membrane module, while a gas mixture of CO_2_/N_2_ flows through the tube side, as depicted in Figure 1. The schematic representations illustrate both ascending and descending spiral ring pitch operations. Two different spiral ring pitches were utilized: ascending pitches of 1 cm to 3 cm and 2 cm to 3 cm in Figure 1a,b, and descending pitches ranging from 3 cm to 1 cm and 3 cm to 2 cm in Figure 1c,d. Device performances were evaluated and compared with those in previous work [33] using constant spiral ring pitches.

### 2.1. Mass Transfer

**Figure 2:**
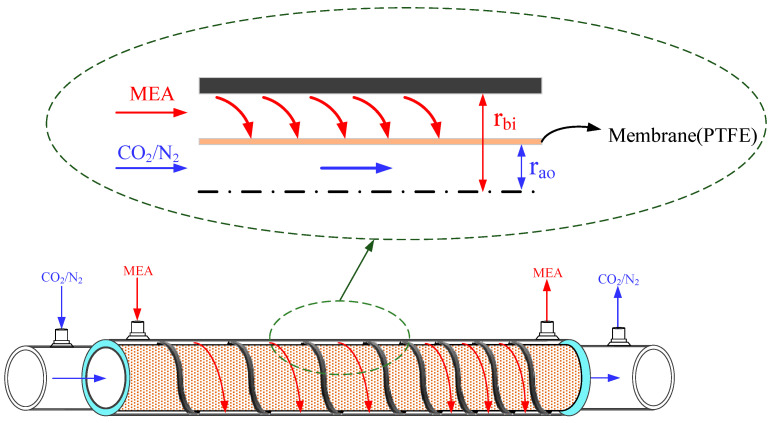
Three regions considered for modeling CO_2_ absorption in a spiral-ring membrane module.

A representation of the mass transfer in the membrane contactor is depicted in Figure 2. $C_{a(g)}$ and $C_{b(l)}$ are the bulk concentrations of the gas feed and MEA liquid solution, respectively, and the membrane surface concentration drops to the concentration $C_{1(g)}$ at the membrane–liquid interface lower than the bulk concentration $C_{a(g)}$ while the membrane surface concentration raises to a higher concentration $C_{2(l)}$ than the bulk concentration $C_{b(l)}$. Henry’s law defined by the dimensionless Henry’s law constant $H_{c}=C_{\left(g\right)}/C_{\left(l\right)}=1.32$ [20] is expressed in terms of the solubility of a gas in a liquid according to the equilibrium with the concentration of the gas in the liquid, or

(1) $$P_{2(g)}=C_{2(g)}RT=hC_{2(l)}$$

or

(2) $$H_{c}=\frac{h}{RT}=\frac{C_{2\left(g\right)}}{C_{2\left(l\right)}}$$

The mathematical modeling equations considered the isothermal diffusion–reaction process occurring within the lumen tube and chemical reactions at the membrane surface on the shell side. These equations were derived to analyze CO_2_ absorption rates in the concentric circular membrane contactor module. The trans-membrane mass flux of CO_2_ is primarily governed by the concentration difference across the membrane, influenced by concentration boundary layers in both bulk streams, membrane properties, and operating conditions. The diffusion–reaction mechanism in the gas/liquid membrane contactor involves three key regions for CO_2_ transfer from the gas feed to the liquid feed: (a) from the bulk gas phase to the membrane surface, (b) through the membrane via its pores, and (c) absorption by the MEA absorbent along with chemical reactions.

The absorption flux $J_{m}$ according to the dusty gas model [14] depends [38] on the trans-membrane saturation partial pressure differences (ΔP) [39] at the membrane surface in the MEA absorbent solution and CO_2_/N_2_ gas side as well as the membrane permeation coefficient ($c_{m}$) [40] as represented in Equation (3)

(3) $$J_{m}=c_{m}(P_{1}-P_{2})\frac{1}{M_{w}}={\left.c_{m}\frac{dP}{dC}\;\right|}_{C_{mean}}(C_{1(g)}-C_{2(g)})\frac{1}{M_{w}}\;=c_{m}RT(C_{1(g)}-{H_{c}K}_{ex}^{\prime }C_{2\left(l\right)})\frac{1}{M_{w}}=K_{m}(C_{1(g)}-{H_{c}K}_{ex}^{\prime }C_{2\left(l\right)})$$

(4) $$K_{ex}^{\prime }=K_{ex}[\mathrm{MEA}]/[H^{+}],K_{ex}=[\mathrm{MEACO}O^{-}]\;[H^{+}]/[CO_{2}][\mathrm{MEA}]=1.25\times 10^{-5}$$

(5) $$c_{m}={\left(\frac{1}{c_{K}}+\frac{1}{c_{M}}\right)}^{-1}={\left\{{\left[1.064\frac{\varepsilon \;r_{p}}{\tau \delta_{m}}{\left(\frac{M_{w}}{RT_{m}}\right)}^{1/2}\right]}^{-1}+{\left[{\left|Y_{m}\right|}_{ln}\frac{D_{m}\varepsilon }{\delta_{m}\tau }\frac{M_{w}}{RT_{m}}\right]}^{-1}\right\}}^{-1}$$

in which $K_{m}$ is the overall mass transfer coefficient of the membrane, $K_{ex}^{\prime }$ is the reduced equilibrium constant with the equilibrium constant $K_{ex}$ at T=298 K [41], and the tortuosity τ=1/ε was determined [42].

The mass diffusion of CO_2_ is transported by the concentration driving force gradient between both gas and liquid bulk streams and membrane surfaces, respectively, as represented below:

(6) $$J_{g}=k_{a}\left(C_{a\left(g\right)}-C_{1\left(g\right)}\right)$$

(7) $$J_{l}=k_{b}\left(K_{ex}^{\prime }C_{2\left(l\right)}-C_{b\left(l\right)}\right){=k}_{L}\left(K_{ex}^{\prime }H_{c}C_{2(l)}-{H_{c}C}_{b(l)}\right)$$

**Figure 3:**
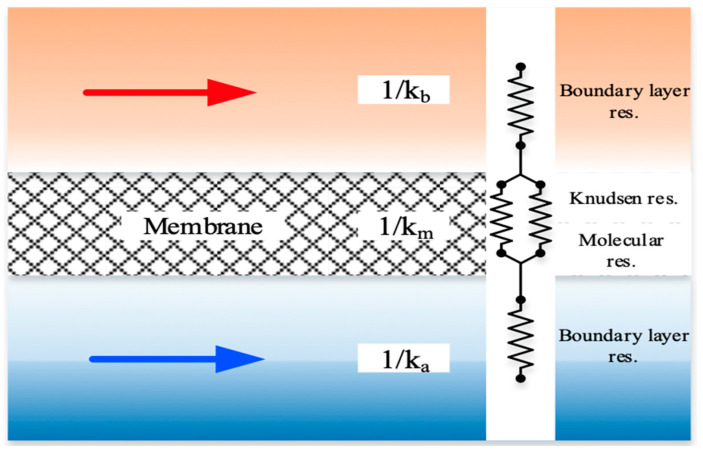
Mass transfer resistances in a gas–liquid membrane contactor.

The individual mass transfer coefficient, based on a resistance-in-series model, in the gas feed ($k_{a}$), membrane ($K_{m}$), liquid feed ($k_{L}=k_{b}/H_{c}$), and CO_2_ concentration variations are illustrated in Figure 3. Equating the mass fluxes by the conservation law of mass in each region for three intervals, ($C_{a\left(g\right)}-C_{1\left(g\right)}),\;(C_{1\left(g\right)}-C_{2\left(g\right)}),$ and ($C_{2(l)}-C_{b(l)}),$ as shown in Equation (8), leads to the overall heat transfer coefficient of the gas feed stream and MEA feed stream, respectively.

(8) $$J_{i}=J_{g}=J_{m}=J_{l}\;i=spiral-ring\;channel\;or\;empty\;channel$$

### 2.2. Concentration Polarization Coefficient

**Figure 4:**
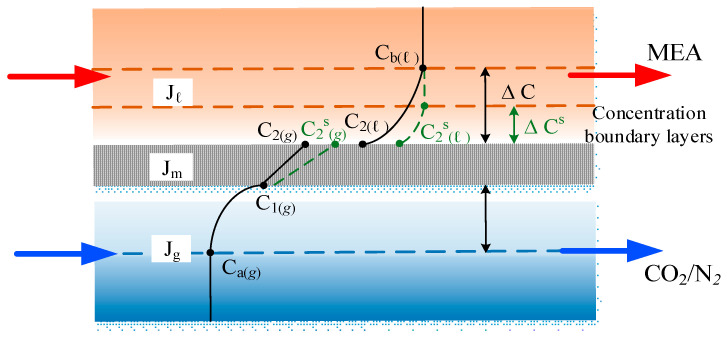
Microscopic description of concentration boundary layers in the membrane contactor module.

The concentration differences between two membrane surfaces associated with two gas and MEA bulk streams, respectively, result in the concentration polarization effect. Various factors govern the mass transfer resistance in membrane absorption modules. The concentration polarization coefficient $\gamma_{m}$ can be considered as a measure of the relative impact of the magnitude of the membrane mass transfer resistance, as indicated in Figure 4. However, the concentration polarization effect could be diminished when turbulence promoters are introduced. The concentration polarization coefficient $\gamma_{m}$ is defined as follows:

(9) $$\gamma_{m}=\frac{C_{1\left(g\right)}-{H_{c}K}_{ex}^{\prime }C_{2(l)}}{C_{a\left(g\right)}-H_{c}C_{b(l)}}=\frac{k_{a}k_{b}}{k_{a}k_{b}+k_{m}k_{b}+k_{m}k_{a}}$$

Accordingly, both membrane surface concentrations ($C_{1(l)}$ and $C_{2(g)}$) and the convective mass transfer coefficients ($k_{a}$ and $k_{b}$) were obtained by equating Equations (3) and (6) (say $J_{m}=J_{g}$) as well as Equations (3) and (7) (say $J_{m}=J_{l}$), respectively. The concentration polarization effect was reduced and demonstrated by using the microscopic description in Figure 4 with the implementation of a spiral-ring channel resulting in the amplified driving force concentration gradient (say $∆C^{s}=C_{2(l)}^{s}-C_{b(l)}$). Implementing spiral-ring channels was confirmed to achieve a higher permeate flux owing to disrupting the mass transfer boundary layer of flow characteristics near the membrane surface, where the intensive turbulence was strengthened to overwhelm the concentration polarization effect.

### 2.3. Design of Spiral-Ring Module

**Figure 5:**
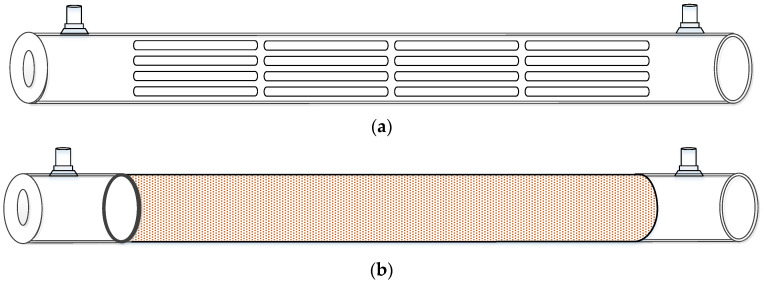

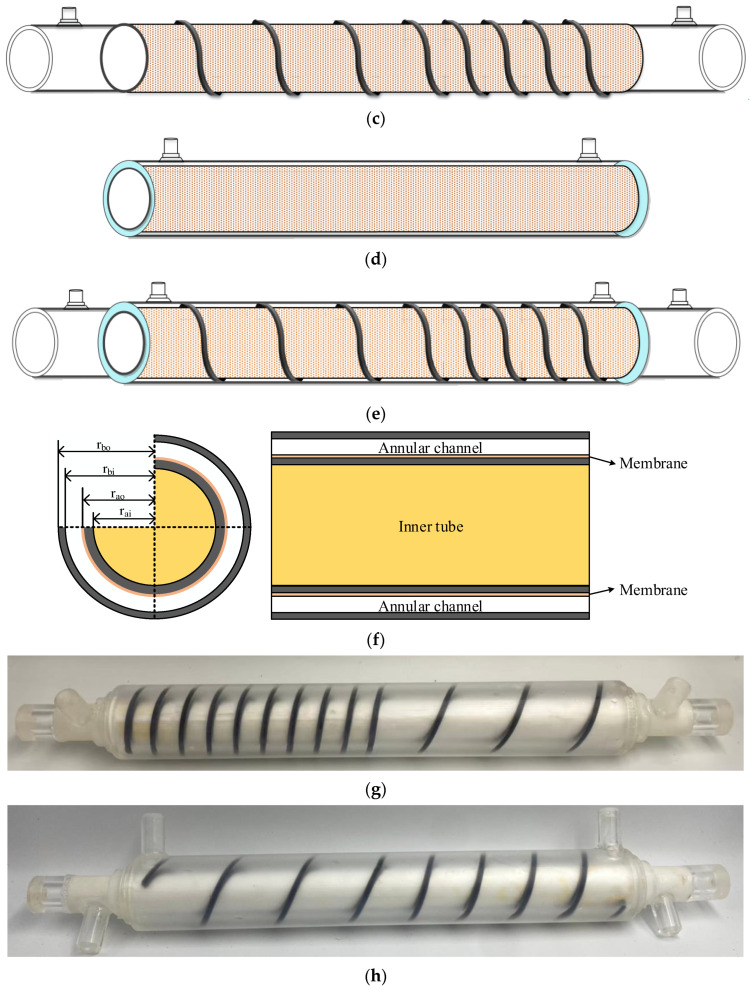
Representative detailed components of the spiral-ring concentric membrane contactor. (**a**) Perforated acrylic lumen tube. (**b**) Wrapped PTFE membrane on the acrylic lumen tube. (**c**) Winding spiral-ring rod on PTFE membrane (say descending 3 mm to 1 mm pitch). (**d**) Outer tube. (**e**) Descending spiral ring pitches from 3 mm to 1 mm. (**f**) Side-view of various radii of inner tube and outer tube. (**g**) Photographic image of membrane tube with 1 cm to 3 cm spiral wire pitches. (**h**) Photographic image of membrane tube with 3 cm to 2 cm spiral wire pitches.

The experimental setup involved detailed fabrication of the spiral ring embedded within the annulus of a concentric circular tube, as illustrated in Figure 5. Turbulence intensity enhancement was achieved by integrating spiral rings as eddy promoters with various spiral ring pitches into the flow channel, effectively disrupting mass transfer boundary layers near the membrane surface within the MEA absorbent feed stream. This approach reduced concentration polarization and led to improvements in convective heat transfer coefficients and overall device performance. Figure 5 provides a graphical representation of key components of the spiral-ring concentric membrane contactor. The experimental setup included an empty channel (without an embedded spiral wire) consisting of a 0.2 m long concentric tubular spiral-ring tube. Spiral ring pitches were fabricated using a photopolymer (light-activated resin), demonstrating stability without degradation during operational experimental runs. The spiral ring-filled annulus channel featured spiral rings with a cross-sectional area of 2 mm × 2 mm, serving as eddy promoters with varying spiral wire pitches. Both ascending (ranging from 1 cm to 3 cm and 2 cm to 3 cm baffled ring distances) and descending (ranging from 3 cm to 1 cm and 3 cm to 2 cm baffled ring distances) spiral ring pitches were tested with MEA flowing through channels of lengths 0.63 m and 0.39 m, respectively. The inner lumen tube, made of light-activated photopolymer resin (He-Yi Precision Co., Taoyuan, Taiwan), had an inside diameter of 1.3 cm and outside diameter of 1.5 cm. It was fabricated using 3D printing technology, which enabled up to 70% perforating holes, a significant advancement compared to traditional manufacturing methods [43], utilizing a layer-by-layer machining process as depicted in Figure 5a. This highlights the capability of 3D printing to produce complex geometric shapes for tubular conduits [44], tailored precisely to the manufacturing process requirements. The inner lumen tube was wrapped with a hydrophobic PTFE/PP composite membrane (J020A330R, ADVANTEC, Toyo Roshi Kaisha, Ltd., Tokyo, Japan) featuring a nominal pore size of 0.2 µm to facilitate gas diffusion through the membrane. It possesses a porosity of 0.72 and a thickness of 130 µm (PTFE 98 µm and PP 32 µm), as depicted in Figure 5b. The spiral ring-filled channel was created by embedding concentric spiral-ring rods around the circumference of the membrane surface on the outer side of the inner lumen tube. These rods were arranged with ascending or descending baffled ring distances, as illustrated in Figure 5c. For the MEA feed stream, the annulus channel dimensions were constructed using an effective 0.2 m long concentric tubular acrylic tube with an inside diameter of 1.9 cm and an outside diameter of 3.0 cm (with a channel thickness of 2 mm), as indicated in Figure 5d. In Figure 5e, the spiral-ring annulus channel, utilizing descending spiral ring pitches such as 3 cm to 1 cm, is displayed. This configuration demonstrates various inner and outer tube radii used in modeling CO_2_ absorption in concentric membrane contactors, illustrated in Figure 5f. Photographic images in Figure 5g,h showcase the membrane tube with spiral wire pitches of 1 cm to 3 cm and 3 cm to 2 cm, respectively. These visuals provide a clear representation of the experimental setup and configuration used in the study.

### 2.4. Model Building by Using Macroscopic Description

**Figure 6:**
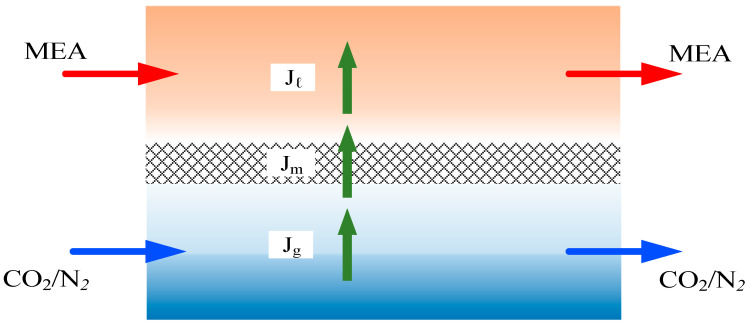
Plug flow description for both CO_2_/N_2_ and MEA feed streams.

The one-dimensional mathematical modeling equations were derived by presenting the mass flux diagram in a finite control element according to the conservation law under steady-state operations, as illustrated by following the schematic diagram of plug flow description in Figure 6 for both CO_2_/N_2_ and MEA feed streams, respectively.

(10) $$\frac{dC_{a}}{dz}=-\frac{2\pi r_{ao}}{q_{a}}\left[k_{m}\gamma_{m}\left(C_{1}-H_{c}{K_{ex}^{\prime }C}_{2\left(l\right)}\right)\right]\;=-\frac{2\pi r_{ao}}{q_{a}}\left[k_{m}\gamma_{m}\left(C_{a\left(g\right)}-H_{c}C_{b\left(l\right)}\right)\right]$$

(11) $$\frac{dC_{b}}{dz}=\frac{2\pi r_{ao}}{q_{b}}\left[k_{m}\gamma_{m}\left(C_{a(g)}-H_{c}C_{b(l)}\right)\right]-\frac{k_{{CO}_{2}}\left[C_{b}\pi \left(r_{bi}^{2}-r_{ao}^{2}\right)\right]}{q_{b}}$$

Equations (10) and (11) were derived by using the mass balances with the plug flow description for CO_2_ absorption in the MEA absorbent stream while the coordinate along *z* is the flowing direction. The plug flow description deals with only the largest concentration gradient in the mass transfer balance equation while neglecting all diffusion terms. The numerical scheme was solved using the 4th order Runge–Kutta method along the module’s length, while the marching solutions of the CO_2_ concentrations in both CO_2_/N_2_ and MEA feed streams were obtained to determine the CO_2_ absorption flux and absorption flux improvement as well.

### 2.5. Assessment of Mass Transfer Rate Enhancement

The assessment of mass transfer enhancement using spiral-ring concentric circular membrane contactors was carried out and evaluated using ascending or descending spiral ring pitches and compared to the no-spiral-ring condition. A mass transfer enhancement factor $\alpha^{S}$ [29] was defined as the ratio of the mass transfer rate improvement of the module with the embedded spiral ring to that of the module without a spiral-ring channel, which depends on inserting various spiral ring pitches and operating different flow configurations. The mass transfer coefficient was analyzed based on the extent of the mass transfer enhancement factor, which was lumped into the augmented mass transfer coefficients and expressed in terms of the effective Sherwood number as follows:

(12) $${Sh}_{spiral}=\frac{k_{b}D_{h,spiral}}{D_{b}}=\alpha^{s}{Sh}_{lam}$$

in which the effective Sherwood number ${Sh}_{spiral}$ is defined as the module of inserting varying-spiral-ring-pitch channels while incorporating four dimensionless groups into Buckingham’s π theorem while the Sherwood number ${Sh}_{lam}$ is the membrane contactor with the no-spiral-ring channel under laminar flow operations, with the regressed correlation equation [45] as:

(13) $$\alpha^{S}=\frac{{Sh}_{spiral}}{{Sh}_{lam}}=f\left(\frac{D_{h,empty}}{L},\frac{D_{h,spiral}}{L},{Re}_{l},{Re}_{g},\;{\;Sc}_{g}\right)$$

in which

(14) $${Sh}_{lam}=3.881{\left(\frac{D_{h,empty}}{L}\right)}^{0.6184}{Re}_{l}^{0.5827}{Re}_{g}^{-0.9322}{Sc}_{g}^{0.9699}$$

where $D_{h,spiral}$ is the equivalent hydraulic diameter of the inserted spiral rings while $D_{h,empty}$ is the hydraulic diameter for laminar flow (no-spiral-ring channel).

### 2.6. Power Consumption Increment

This present study offers a possible investigation to utilize spiral-ring channel designs as turbulence promoters for membrane contactor applications. The individual in-depth impact contributes towards device performance improvement by using the spiral ring-filled channels along with the extra unavoidable power consumption. Further focused research based on economic consideration is needed to report the influence of spiral-ring channels. There is a need for specific understanding to evaluate the increased frictional loss by employing a spiral ring-filled channel in the concentric-tube membrane contactor module. The power consumption includes the contributions from both the gas side and MEA side for the innovative spiral ring-filled channel design, which can be developed by using the Fanning friction factor $f_{F}$ for both laminar and turbulent flows [46]:

(15) $$H_{i}=q_{a}\;\rho_{{CO}_{2}}\;lw_{f,{CO}_{2}}+q_{b}\;\rho_{MEA}\;lw_{f,MEA}\;i=spiral,\;empty$$

(16) $${lw}_{f,MEA}=\frac{2f_{F,MEA}{\bar{v}}_{MEA}^{2}L}{D_{h,MEA}}$$

(17) $${lw}_{f,{CO}_{2}}=\frac{2f_{F,{CO}_{2}}{\bar{v}}_{{CO}_{2}}^{2}L}{D_{h,{CO}_{2}}}$$

This quantity comprises the pressure drop due to the friction losses in both feed streams, as given by Equation (15), in a concentric circular membrane contactor of known length. The average velocity and equivalent hydraulic diameter of each flow channel are calculated as follows:

(18) $${De}_{h,CO_{2}}=2r_{ai},\;{\bar{v}}_{CO_{2}}=\frac{q_{a}}{\pi r_{ai}^{2}\;}$$

(19) $${De}_{h,MEA}=\frac{4\left[W_{p}\left(r_{bi}-r_{ao}\right)\right]}{2\left[W_{p}\left(r_{bi}-r_{ao}\right)\right]},\;{\bar{v}}_{MEA}=\frac{q_{b}}{W_{p}\left(r_{bi}-r_{ao}\right)}$$

The percentage increment in power consumption for the module with the spiral ring-filled channel as compared to the module with an empty channel is illustrated as the relative extents $I_{p}$

(20) $$I_{H}=\frac{H_{spiral}-H_{empty}}{H_{empty}}\times 100\%$$

where the subscripts of *spiral* and *empty* represent the modules using the spiral ring-filled channel and empty channel, respectively.

## 3. Membrane Absorption Experiments

**Figure 7:**
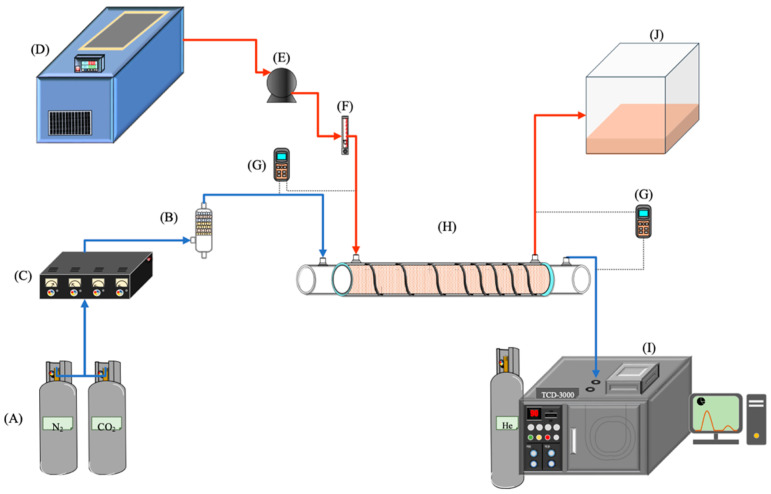
Scheme of a setup used in experiments of concentric membrane contactor modules (A—gas cylinder; B—gas mixing cylinder; C—mass flow controller; D—thermostatic tank; E—pump; F—flow meter; G—thermometer; H—membrane absorption module; I—chromatograph; J—MEA collector).

**Figure 8:**
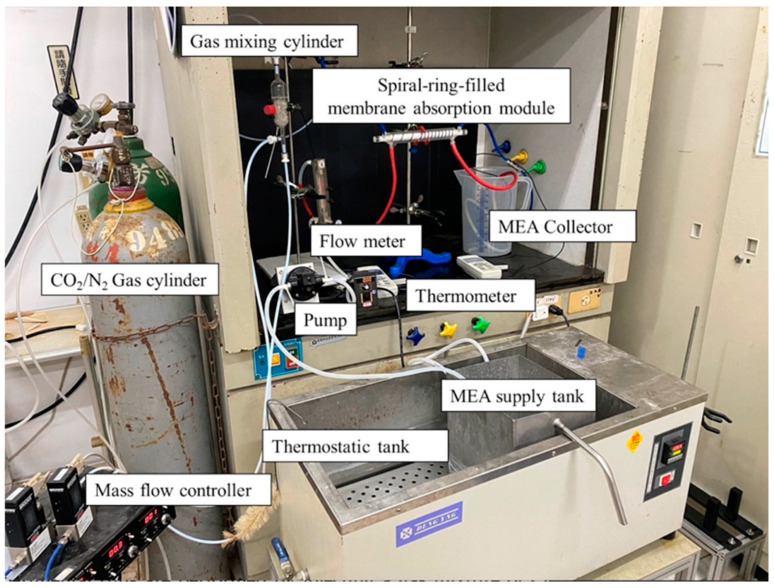
Photographic images of the experimental apparatus for the concentric spiral ring pitch module.

The gas/liquid membrane contactor for CO_2_ absorption by the MEA absorbent (Uni-Onward Corp., New Taipei, Taiwan) was conducted in an acrylic concentric tube scale setup, as illustrated in Figure 7. A photo of the operating experimental apparatus is shown in Figure 8.

The experimental setup involved injecting a gas mixture of CO_2_/N_2_ at 293 K into the inner lumen tube of a membrane absorption contactor, while the MEA absorbent solution flowed through the annulus channel. The aqueous MEA absorbent solution was heated to 303 K using a thermostat (G-50, DENG YNG, New Taipei, Taiwan) and controlled by a flow meter (MB15GH-4-1, Fong-Jei, New Taipei, Taiwan) and liquid pump (5IK40RA-A, ASTK Motor Co. Ltd., New Taipei, Taiwan) to regulate flow rates ranging from 5 to 10 cm^3^/s (5.0, 6.67, 8.33, 10.0 cm^3^/s). The MEA absorbent was prepared by diluting it with distilled water to a 30% mass concentration and it was then introduced into the shell side of the contactor at various feed rates. After contacting with the CO_2_/N_2_ gas mixture, the MEA absorbent containing CO_2_ was collected separately. Industrial-grade gas mixtures containing 30%, 35%, and 40% CO_2_ (balance N_2_) were mixed in a gas mixing tank (EW-06065-02, Cole Parmer Company, Vernon Hills, IL, USA) and adjusted using a mass flow controller (N12031501PC-540, Protec, Brooks Instrument, Hatfield, PA, USA) to maintain a steady flow rate of 5 cm^3^/s into the inner lumen side of the membrane module. The CO_2_ diffused through the microporous hydrophobic membrane pores into the MEA absorbent on the shell side of the contactor. All experiments were conducted with concurrent flows and single-throughput operations. The CO_2_ exiting the membrane lumen-side outlet was sampled and injected into column heating systems for rapid sample heating within a collection capillary tube. Gas chromatography (Model HY 3000, China Chromatograph Co., Ltd., Xinzhuang, New Taipei, Taiwan) with helium as a carrier gas and conventional thermal conductivity detectors (TCD) were used to analyze CO_2_ concentrations at steady state. The reproducibility of concentration measurements was monitored, ensuring accuracy within 5% to determine CO_2_ absorption efficiency.

## 4. Results and Discussions

### 4.1. Correlated Sherwood Numbers

**Figure 9:**
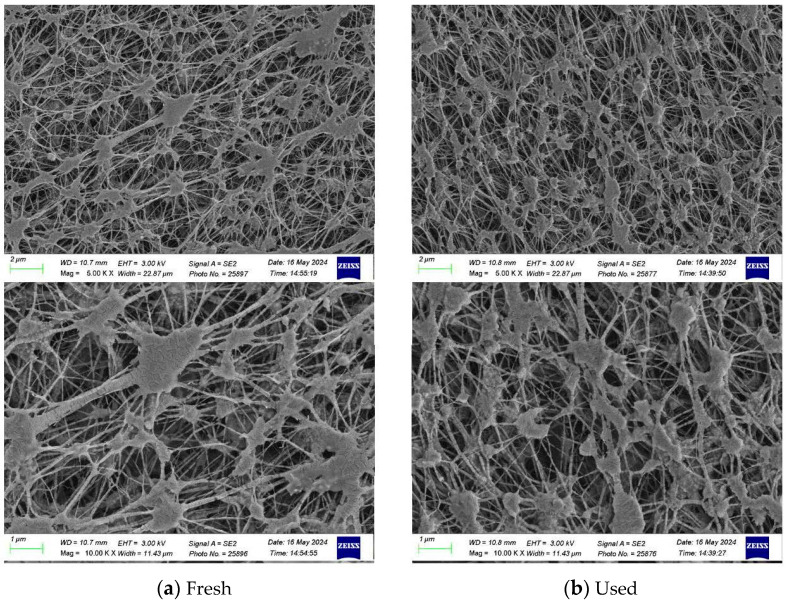
SEM images of the PTFE/PP membrane for fresh and used membranes of experimental runs.

**Figure 10:**
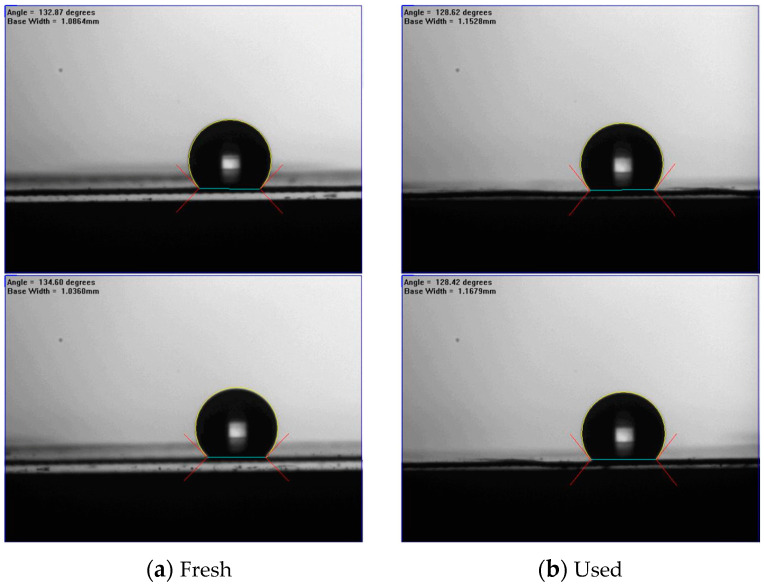
Sessile-drop contact angles of PTFE/PP membranes.

The morphology and water contact angles of the composite membranes (PTFE/PP) before and after experimental runs were tested and are presented in Figure 9 and Figure 10. Figure 9 shows the morphology by using a scanning electron microscopic (SEM, Zeiss sigma 300, Jena, Germany) on the fresh and used membranes of experimental runs while the water contact angles of the PTFE/PP membranes were determined (First Ten Angstrom FTA-125, Portsmouth, NH, USA) to be in the range of 128–134° (water contact angle of 131.2 ± 3.0°) to confirm the surface hydrophobicity of the hydrophobic membrane, as shown in Figure 10.

The range and limits of deviation between theoretical predictions and experimental results for all measurements are calculated using the following definition of accuracy deviation [47]:

(21) $$E\;(\%)=\frac{1}{N_{exp}}\sum_{j=1}^{N_{exp}}\frac{\left|J_{theo,j}-J_{\exp,j}\right|}{J_{\exp,j}}\;$$

where $N_{exp}$, $J_{theo,j}$, and $J_{\exp,j}$ are the number of experimental runs, theoretical predictions, and experimental results of absorption fluxes, respectively. The accuracy deviations of both ascending and descending spiral ring pitches are shown in Table 1 and Table 2 for illustration. The agreement of experimental results deviated from theoretical predictions is fairly good, within $4.0\times 10^{-4}\leq E\leq 4.87\times 10^{-2}$.

**Figure 11:**
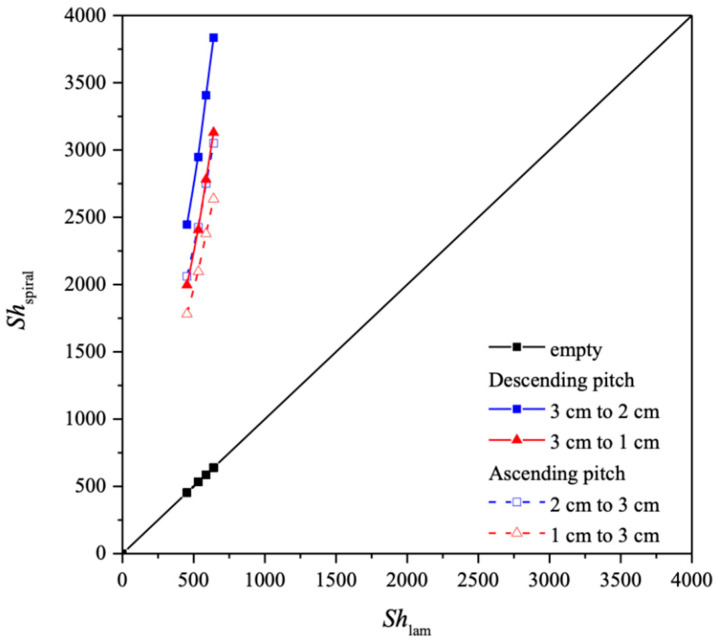
Comparison of correlated and experimental Sherwood numbers for the no-spiral-ring channel and spiral ring-filled channel under both descending and ascending spiral ring pitches.

Application of Runge–Kutta numerical scheme in a marching solution procedure to Equations (10) and (11) was performed to obtain the CO_2_ concentration distributions in the CO_2_/MEA bulk streams as well as CO_2_ absorption flux for various spiral ring pitches. Comparisons were made for the CO_2_ absorption flux of modules using the spiral ring-filled channel and the no-spiral-ring channel under both ascending and descending spiral ring pitch operations. The mass transfer coefficients expressed in terms of the Sherwood number, as presented in Equation (12), were determined by the theoretical model in comparison with the module with no-spiral-ring operations, as shown in Figure 11. The correlated Sherwood numbers for the device with the no-spiral-ring channel are in linear consistency with the experimental data, as referred to in Equation (14).

The influence of descending and ascending spiral ring pitches with various configurations on the Re and Sh numbers were derived from the correlation via a regression analysis. The Sherwood numbers for the no-spiral-ring channel are in a linear diagonal straight line with the experimental data, as shown in Figure 11, while the correlation expression of Sherwood numbers is also applicable for the channel with embedded spiral ring-filled turbulence promoters. The Sherwood number in the channels with inserted spiral ring-filled turbulence promoters is higher than that of the module with a no-spiral-ring channel, which results in the augmented convective mass transfer coefficients in membrane absorption modules presented in Equations (22) and (23), as well as Figure 11. A regression analysis was set up with the normal equations for the least square parameters to obtain the correlated equation as follows.

(22) $${Sh}_{spiral}=3.574{W_{r}^{0.2582}(\frac{D_{h,spiral}}{L})}^{0.5389}{Re}_{l}^{0.6489}{Re}_{g}^{4.213}{Sc}_{g}^{1.685}\;\mathrm{Descending}\;\mathrm{pitches}$$

(23) $${Sh}_{spiral}=6.029W_{r}^{0.2379}{\left(\frac{D_{h,spiral}}{L}\right)}^{0.6032}{Re}_{l}^{0.565}{Re}_{g}^{-0.3325}{Sc}_{g}^{1.36}\;\mathrm{Ascending}\;\mathrm{pitches}$$

in which $W_{r}$ is the ratio of various pitches inserted.

**Figure 12:**
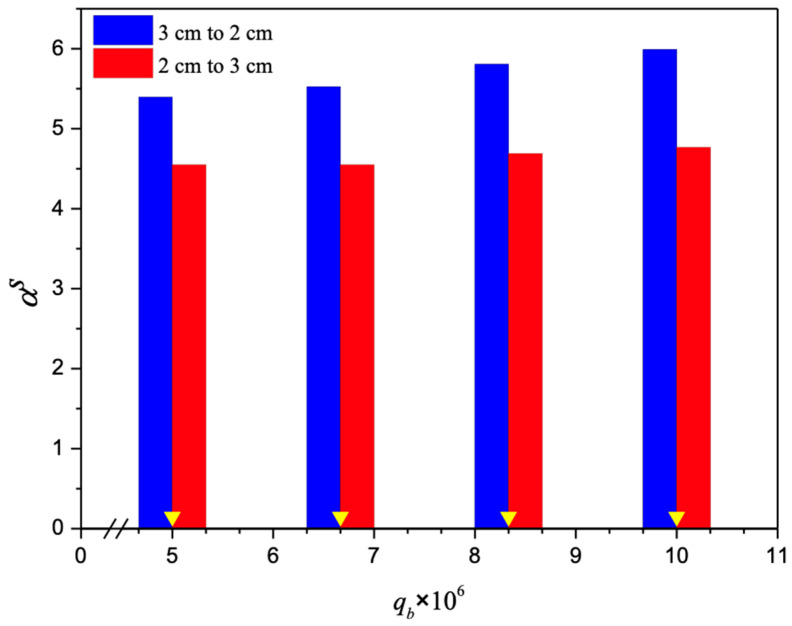
Comparisons of theoretical Sherwood numbers for the channels with inserted spiral ring-filled turbulence promoters (The yellow triangles indicate volumetric flow rates).

The absorption fluxes were calculated through the mass transfer enhancement factor with the inserted spiral ring-filled turbulence promoters in the flow channel with descending and ascending pitches, Equations (22) and (23), for predicting the Sherwood numbers, referred to as the mass transfer efficiency, respectively. Another aspect that could be further examined is the directionality of ascending or descending spiral ring pitches relative to the MEA flow stream direction. The improved performance of the descending spiral ring pitch operations in comparison to that of the ascending spiral ring pitch operations is a good indication of the influence on device performance due to the induced turbulence intensity. Hence, the flow disruptive manner of the mass transfer boundary layer improves performance by creating turbulence intensity. Furthermore, the correlated Sherwood number was incorporated into the mass transfer enhancement factor $\alpha^{s}$ of Equation (13), as the improved mass transfer coefficient reduces the concentration polarization effect, and thus the driving force across the membrane as well as the absorption flux is increased. The mass transfer enhancement factor was derived from the correlation via a regression analysis and expressed in Equation (13) for implementing the spiral ring-filled channel, which results in the augmented convective mass transfer coefficients in the membrane absorption modules, as presented in in Figure 12.

**Figure 13:**
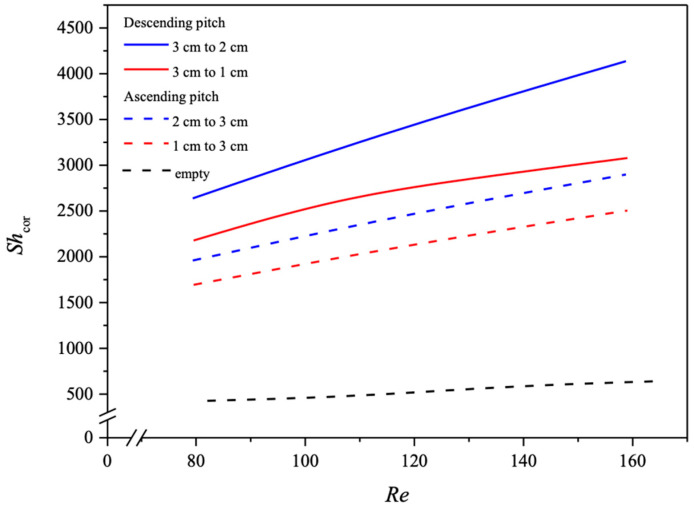
Effects of both descending and ascending spiral wire pitches on Sherwood numbers.

The correlated Sherwood numbers, as shown in Figure 13, indicate that the mass transfer coefficient of the device with descending spiral ring-filled channels accomplishes a higher mass transfer rate than that of the ascending spiral ring-filled channels and the no-spiral-ring channel as well. The spiral ring pitch plays an important role in the mass transfer rate enhancement, which is ascribed to the interruption of the concentration boundary layer, and thus, the CO_2_ absorption flux was increased.

### 4.2. CO_2_ Absorption Flux Improvement by Embedding Various Spiral Ring Pitches

**Figure 14:**
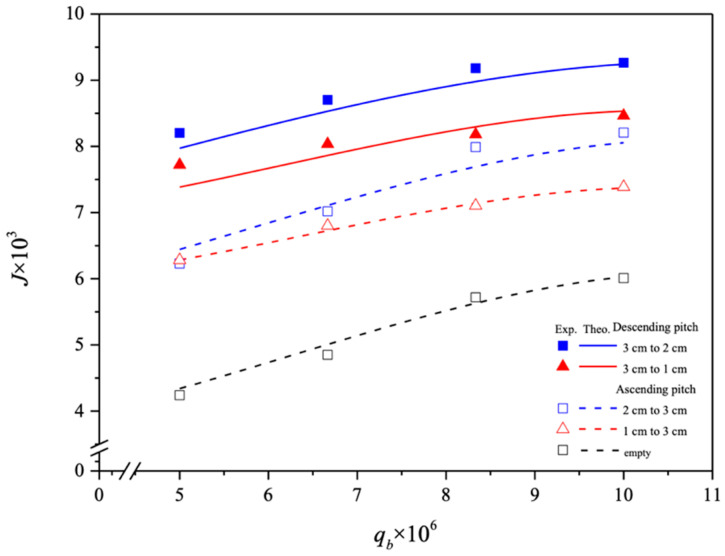
Comparisons of theoretical CO_2_ absorption flux under both ascending and descending spiral ring pitches.

The utilization of the spiral-ring channel significantly enhanced CO_2_ absorption flux performance compared to operating without spiral rings. Introducing various spiral ring pitches increased turbulence intensity and shear near the membrane surface, resulting in a nearly a threefold increase in CO_2_ absorption flux. Figure 14 illustrates that descending spiral ring pitches exhibited superior absorption flux performance compared to ascending pitches. This enhancement is attributed to the reduced mass transfer resistances across the concentration boundary layers in the liquid phase, as observed in the experimental setup [30]. Moreover, all spiral ring pitches were tested under consistent operating conditions of feed flow rates and membrane materials.

**Figure 15:**
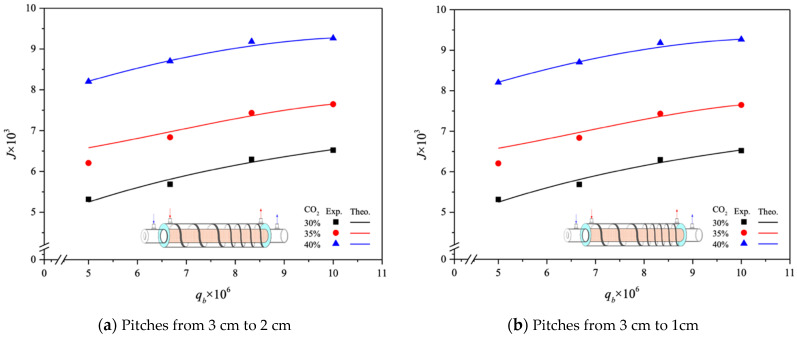
Effects of MEA flow rate and inlet CO_2_ feed concentration on CO_2_ absorption flux under ascending and descending spiral ring pitches.

**Figure 16:**
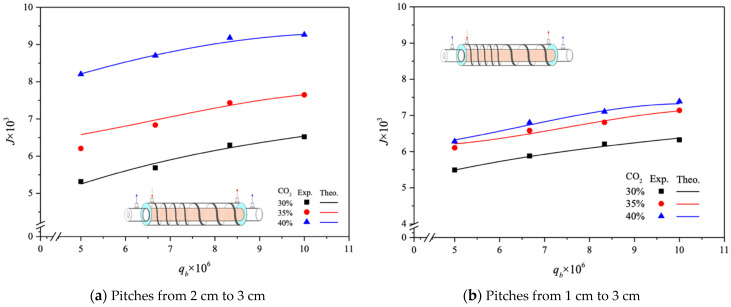
Effects of MEA flow rate and inlet CO_2_ feed concentration on CO_2_ absorption flux under ascending and descending spiral ring pitches.

The CO_2_ absorption fluxes for the module with both ascending and descending spiral ring pitch operations are shown in Figure 15 and Figure 16, including experimental results and theoretical predictions, respectively. In general, the CO_2_ absorption flux with the embedded spiral-ring channels is more substantially improved in descending-pitch operations than that in ascending-pitch operations. As expected, the increase in both MEA feed flow rate and inlet feed CO_2_ concentration yields a higher absorption flux.

The absorption flux improvements $I_{E}^{as}$ and $I_{E}^{des}$ were illustrated by calculating the percentage increase in the device with ascending and descending spiral ring pitches, respectively, based on the device with the no-spiral-ring channel, as

(24) $$I_{E}^{as}(\%)=\frac{J_{spiral}^{as}-J_{empty}}{J_{empty}}\times 100,\;\mathrm{module}\;\mathrm{with}\;\mathrm{ascending}\;\mathrm{pitches}$$

(25) $$I_{E}^{des}(\%)=\frac{J_{spiral}^{des}-J_{empty}}{J_{empty}}\times 100,\;\mathrm{module}\;\mathrm{with}\;\mathrm{descending}\;\mathrm{pitches}$$

where the subscripts *spiral* and *empty* represent the channels with/without inserted spiral rings, respectively, and the superscripts *as* and *des* represent ascending and descending spiral ring pitch operations, respectively. The theoretical predictions of the CO_2_ absorption flux improvements $I_{E}^{as}$ and $I_{E}^{des}$ for various MEA feed flow rates and inlet feed CO_2_ concentrations under ascending and descending spiral ring pitch operations are summarized in Table 3 and Table 4, respectively.

The relative increments in absorption flux improvements, $I_{E}^{as}$ and $I_{E}^{des}$, were calculated by comparing the absorption flux in modules with spiral ring-filled channels to those without spiral rings. Table 3 and Table 4 highlight that the maximum absorption flux improvement reached up to 83.69% under 40% inlet CO_2_ concentration and a 5 cm^3^/s MEA feed flow rate compared to the no-spiral-ring module condition. Previous research [33] demonstrated superior device performance with smaller spiral ring pitches under constant spiral ring-filled channels; specifically, the 2 cm module outperformed the 3 cm module, as well as both the 3 cm to 2 cm and 2 cm to 3 cm modules. It is noteworthy that operating the descending 3 cm to 1 cm spiral ring-filled channel generally resulted in higher absorption flux improvements compared to the descending 3 cm to 2 cm channel, especially evident at higher inlet CO_2_ concentrations (e.g., 40%), as confirmed by Table 4. Conversely, for the ascending 1 cm to 3 cm and 2 cm to 3 cm spiral ring-filled channels, there was minimal relative variance in absorption flux improvements, as shown in Table 3, indicating a slight concentration polarization effect in the first half of the module. Overall, inserting spiral rings into the flow channel demonstrates significant potential to enhance absorption flux, with more substantial improvements observed in descending spiral ring pitch operations compared to ascending ones.

### 4.3. Further CO_2_ Absorption Flux Enhancement

**Figure 17:**
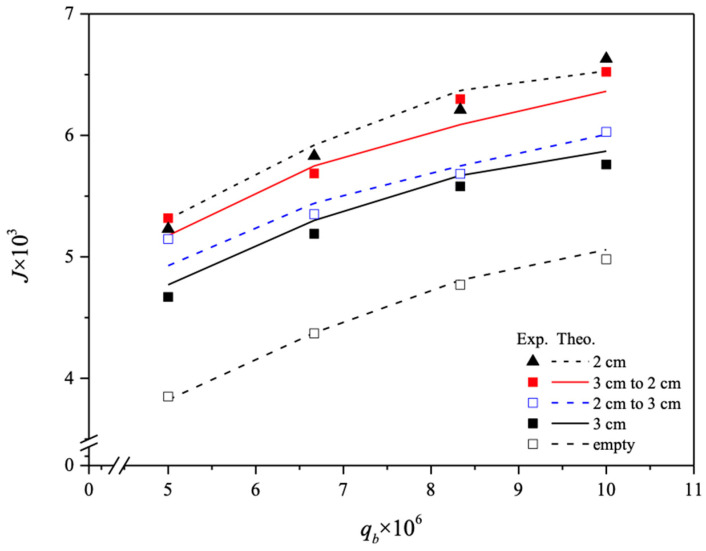
Comparisons of theoretical CO_2_ absorption flux with/without inserting turbulence promoters under various spiral ring pitches [33].

This current study builds upon our previous research [33] by exploring varying-spiral-ring-pitch channels in both ascending and descending operations, as depicted in Figure 17. This approach highlights the technical feasibility of this approach and demonstrates significantly enhanced absorption flux compared to our earlier work [33], i.e., where the performance of a 2 cm module is better than that of a 3 cm module, as well as that of both 3 cm to 2 cm and 2 cm to 3 cm modules. Concentration polarization increases notably in the latter half of the module, leading to higher absorption flux in modules using descending 3 cm to 2 cm spiral ring-filled channels compared to those employing ascending 2 cm to 3 cm and constant 3 cm spiral ring-filled channels. Figure 17 illustrates the order of absorption fluxes among membrane absorption modules utilizing spiral ring-filled channels: 2 cm > 3 cm to 2 cm > 2 cm to 3 cm > 3 cm.

The descending spiral ring pitches continued to be the better performing of the varying-spiral-ring-pitch channels, with a higher absorption flux than those of the constant spiral ring pitch operations in the previous work [33] as an illustration. The further absorption flux enhancement $E_{spiral}$ of CO_2_ capture in the spiral ring-filled membrane contactors through the implementation of spiral-ring channels under descending spiral ring pitches is calculated based on the device of the same working dimensions performed in the previous work [33] with the constant pitch of 3 cm as follows:

(26) $$E_{spiral}(\%)=\frac{J_{spiral}^{des}-J_{sipral}^{con}}{J_{sipral}^{con}}\times 100=\left[\frac{\left(J_{spiral}^{des}-J_{empty})-(J_{sipral}^{con}-J_{empty}\right)}{J_{empty}}\right]\left(\frac{J_{empty}}{J_{sipral}^{con}}\right)\times 100\;=\left(I_{E}^{des}-I_{E}^{con}\right)/\left(1+I_{E}^{con}\right)\times 100$$

where $J_{sipral}^{con}$ and $J_{spiral}^{des}$ are the absorption fluxes in the module with the constant spiral ring pitch of 3 cm [33] and the varying spiral ring pitches in this present study, respectively. A percentage increment in absorption flux improvement and further absorption flux enhancement was evaluated for the module with both descending spiral ring pitches, i.e., 3 cm to 2 cm and 3 cm to 1 cm, respectively, as seen from Table 5.

Further enhancement in absorption flux is achieved by embedding varying spiral ring pitches into the MEA feed stream, thereby increasing the convective mass transfer coefficient and turbulence intensity. For instance, a maximum of 40.85% enhancement in absorption flux is achieved under similar operating conditions compared to the module with a constant spiral ring pitch of 3 cm, as detailed in Table 5. Furthermore, the enhancement in absorption flux with various spiral ring pitches increases with higher inlet feed CO_2_ concentration but decreases with lower MEA feed flow rates.

### 4.4. Power Consumption Increment

**Figure 18:**
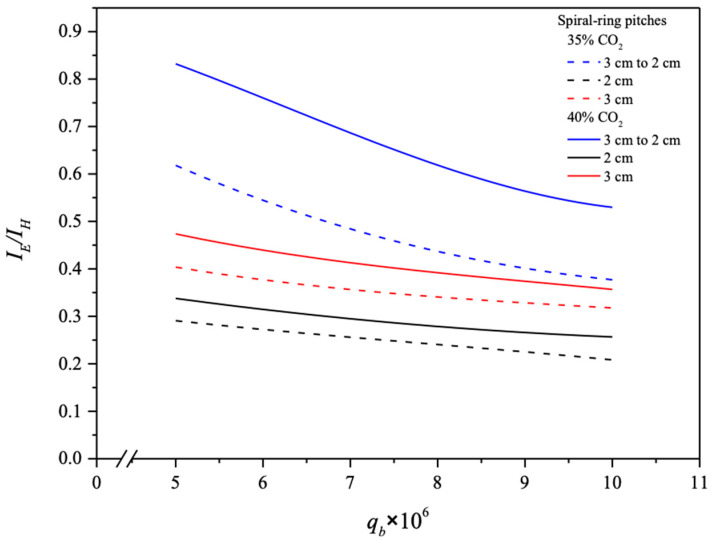
Effects of MEA flow rate and spiral ring pitches on $I_{E}/I_{H}$.

The absorption flux enhancement is compensated by a power consumption increment due to the introduction of spiral rings causing a hydraulic drop in MEA feed pressure, which is attributed to the trade-offs of additional friction losses due to the amplified turbulent intensity as a result of the insertion of tight-fitting spiral rings into a small concentrical annulus. An indicator of the ratio $I_{E}/I_{H}$ on the economic viewpoint in making the suitable selection of operation conditions was delineated and is shown in Figure 18 with MEA flow rates and spiral ring pitches as parameters.

The increase in the CO_2_ absorption flux cannot compensate for the power consumption increment by increasing the MEA flow rate. The results indicate that the descending spiral ring pitches had larger $I_{E}/I_{H}$ values than those of modules with constant spiral ring pitches and ascending spiral ring pitches. Restated, the percentage increment of absorption flux improvement is higher than the percentage of energy consumption increment. In essence, implementing spiral ring pitch turbulence promoters in the MEA absorbent feed stream achieves more absorption flux improvement at the expense of the friction loss increment, as revealed in Figure 18. Notably, the values of $I_{E}/I_{H}$ increase with inlet CO_2_ concentration due to the larger concentration driving force gradients in descending-spiral-ring-pitch systems. A narrower-spiral-ring-pitch channel induces a higher turbulence intensity that results in the mass transfer resistance reduction [33]. The comparison exposes a higher absorption flux in operating the device with a constant 2 cm spiral ring pitch channel than that in the device with a descending 3 cm to 2 cm spiral ring pitch channel, as shown Figure 17. By contrast, the ratio of $I_{E}/I_{H}$ for various spiral ring pitches are in reverse order, especially for both modules with spiral ring pitches of 2 cm and 3 cm to 2 cm. In other words, the descending 3 cm to 2 cm spiral ring pitch operation can increase CO_2_ absorption flux more effectively than the 2 cm spiral ring pitch operation, regarding the assessment on an economic viewpoint, as indicated in Figure 18. The $I_{E}/I_{H}$ ratio for the channel with varying spiral ring pitches exceeds that of the channel with a constant spiral ring pitch, which implies that suitably varying spiral ring pitches can accomplish a larger absorption flux at the expense of energy consumption. This is the value of this present study.

## 5. Conclusions

Theoretical predictions were calculated and validated against experimental results involving various MEA flow rates, inlet feed concentrations, and different spiral ring pitches. Comprehensive demonstrations of the benefits of spiral ring-filled channels with varying pitches in concentric circular membrane absorption modules led to the following conclusions:

1. Embedding spiral ring-filled channels with descending pitches (3 cm to 2 cm) into the MEA flow channel results in a relative increment in absorption flux, achieving a maximum improvement of 83.69% under a 40% inlet CO_2_ concentration and a 5 cm^3^/s MEA feed flow rate.
2. This study shows a higher absorption flux improvement in modules with constant-spiral-ring-pitch channels compared to those with descending 3 cm to 2 cm spiral ring pitches. However, the ratio of $I_{E}/I_{H}$ for varying spiral ring pitches follows a reverse order.
3. Absorption flux improvement is more pronounced in descending spiral ring pitch operations compared to ascending ones due to the achievement of a larger concentration gradient across the entire module.
4. Among the flow characteristics of varying spiral ring pitches, descending pitches exhibit a strong positive influence on absorption flux, attributed to the concentration gradient effectively induced by properly adjusting these pitches.

The correlated equation of the Sherwood number, derived from theoretical modeling, proves invaluable for designing more efficient membrane absorption applications. This paper specifically emphasizes enhancing turbulence intensity in the design of a concentric circular gas/liquid PTFE/PP membrane contactor for CO_2_ absorption, achieved through embedding channels with varying spiral ring pitches.

## Figures and Tables

**Table 1:** The accuracy deviation between theoretical predictions and experimental results.

$C_{in}$	$q_{b}\times 10^{6}$	Ascending Spiral Ring Pitch Operations (mol m^−2^ s^−1^)					
		2 cm to 3 cm			1 cm to 3 cm		
(%)	m^3^/s	$J_{exp}\times 10^{3}$	$J_{theo}\times 10^{3}$	E (%)	$J_{exp}\times 10^{3}$	$J_{theo}\times 10^{3}$	E (%)
30	5.0	5.15	4.93	4.46	5.49	5.66	2.95
	6.67	5.35	5.44	1.67	5.88	5.97	1.40
	8.33	5.68	5.75	1.14	6.21	6.12	1.53
	10.0	6.03	6.01	0.35	6.33	6.26	1.10
35	5.0	5.65	5.74	1.51	6.11	6.11	0.02
	6.67	6.29	6.15	2.21	6.58	6.28	4.87
	8.33	6.81	6.84	0.44	6.81	6.83	0.35
	10.0	7.01	7.13	1.69	7.14	7.00	1.95
40	5.0	6.23	6.44	3.31	6.28	6.28	0.04
	6.67	7.02	7.10	1.17	6.80	6.69	1.68
	8.33	7.99	7.85	1.73	7.11	7.28	2.42
	10.0	8.21	8.06	1.90	7.39	7.37	0.23

**Table 2:** The accuracy deviation between theoretical predictions and experimental results.

$C_{in}$	$q_{b}\times 10^{6}$	Descending Spiral Ring Pitch Operations (mol m^−2^ s^−1^)					
		3 cm to 2 cm			3 cm to 1 cm		
(%)	m^3^/s	$J_{exp}\times 10^{3}$	$J_{theo}\times 10^{3}$	E (%)	$J_{exp}\times 10^{3}$	$J_{theo}\times 10^{3}$	E (%)
30	5.0	5.32	5.18	2.76	5.90	6.15	4.08
	6.67	5.69	5.75	1.08	6.52	6.48	0.60
	8.33	6.30	6.09	3.44	6.83	6.65	2.73
	10.0	6.52	6.36	2.51	6.92	6.80	1.89
35	5.0	6.21	6.59	5.79	6.65	6.86	3.06
	6.67	6.84	7.03	2.81	7.23	7.08	2.03
	8.33	7.43	7.65	2.84	7.46	7.62	2.09
	10.0	7.65	7.90	3.12	7.65	7.79	1.89
40	5.0	8.20	7.97	2.90	7.72	7.38	4.54
	6.67	8.70	8.56	1.65	8.04	7.84	2.53
	8.33	9.18	9.10	0.92	8.18	8.44	3.13
	10.0	9.26	9.24	0.24	8.46	8.53	0.81

**Table 3:** Effects of ascending spiral ring pitch operations on absorption flux improvements.

$C_{in}$	$q_{b}\times 10^{6}$	Ascending Spiral Ring Pitch Operations (mol m^−2^ s^−1^)				
		Empty Channel	2 cm to 3 cm		1 cm to 3 cm	
(%)	(m^3^ s^−1^)	$J_{empty}\times 10^{3}$	$J_{spiral}^{as}\times 10^{3}$	$I_{E}^{as}$ (%)	$J_{spiral}^{as}\times 10^{3}$	$I_{E}^{as}$ (%)
30	5.0	3.82	4.93	28.88	5.66	48.05
	6.67	4.37	5.44	24.42	5.97	36.38
	8.33	4.81	5.75	19.57	6.12	27.20
	10.0	5.06	6.01	18.76	6.26	23.71
35	5.0	4.03	5.74	42.60	6.11	51.65
	6.67	4.60	6.15	33.88	6.28	27.97
	8.33	5.34	6.84	28.18	6.83	25.59
	10.0	5.58	7.13	27.86	7.00	25.59
40	5.0	4.34	6.44	48.46	6.28	44.79
	6.67	4.97	7.10	42.90	6.69	34.58
	8.33	5.80	7.85	35.39	7.28	25.55
	10.0	6.03	8.06	33.63	7.37	22.27

**Table 4:** Effects of descending spiral ring pitch operations on absorption flux improvements.

$C_{in}$	$q_{b}\times 10^{6}$	Descending Spiral Ring Pitch Operations (mol m^−2^ s^−1^)				
		Empty Channel	3 cm to 2 cm		3 cm to 1 cm	
(%)	(m^3^ s^−1^)	$J_{empty}\times 10^{3}$	$J_{spiral}^{des}\times 10^{3}$	$I_{E}^{des}$ (%)	$J_{spiral}^{des}\times 10^{3}$	$I_{E}^{des}$ (%)
30	5.0	3.82	5.18	35.37	6.15	60.85
	6.67	4.37	5.75	31.42	6.48	48.08
	8.33	4.81	6.09	26.64	6.65	38.22
	10.0	5.06	6.36	25.77	6.80	34.32
35	5.0	4.03	6.59	63.64	6.86	70.51
	6.67	4.60	7.03	53.07	7.08	54.09
	8.33	5.34	7.65	43.28	7.62	42.67
	10.0	5.58	7.90	41.59	7.79	39.76
40	5.0	4.34	7.97	83.69	7.38	70.16
	6.67	4.97	8.56	72.26	7.84	57.69
	8.33	5.80	9.10	56.86	8.44	45.59
	10.0	6.03	9.24	53.33	8.53	41.55

**Table 5:** Theoretical predictions of absorption flux improvements and further absorption flux enhancement in the module with descending spiral ring pitches.

$C_{in}$	$q_{b}\times 10^{6}$	Descending Spiral Ring Pitches				
		Constant 3 cm Pitch [33]	3 cm to 2 cm		3 cm to 1 cm	
(%)	m^3^/s	$I_{E}^{con}$ (%)	$E_{spiral}$ (%)	$I_{E}^{des}$ (%)	$E_{spiral}$ (%)	$I_{E}^{des}$ (%)
30	5.0	24.77	8.49	35.37	28.92	60.85
	6.67	21.17	8.46	31.42	22.21	48.08
	8.33	17.93	7.38	26.64	17.21	38.22
	10.0	16.03	8.40	25.77	15.77	34.32
35	5.0	26.92	28.93	63.64	30.23	65.30
	6.67	25.98	21.50	53.07	22.32	54.09
	8.33	23.41	16.10	43.28	15.61	42.67
	10.0	21.41	16.62	41.59	15.11	39.76
40	5.0	30.41	40.85	83.69	36.40	77.89
	6.67	27.36	35.25	72.26	23.81	57.69
	8.33	25.17	25.32	56.86	16.32	45.59
	10.0	24.25	23.40	53.33	13.92	41.55

## Data Availability

The original contributions presented in the study are included in the article, further inquiries can be directed to the corresponding author.

## Abbreviations

C	Concentration (mol m^−3^)
$C_{mean}$	Mean value of C (mol m^−3^)
$c_{K}$	Membrane coefficient based on the Knudsen diffusion model (mol m^−2^ pa^−1^ s^−1^)
$c_{M}$	Membrane coefficient based on the molecular diffusion model (kg m^−2^ Pa^−1^ s^−1^)
$c_{m}$	Membrane permeation coefficient (mol m^−2^ pa^−1^ s^−1^)
$D_{a}$	Diffusion coefficient of CO_2_ in gas feed stream (m^2^ s^−1^)
$D_{b}$	Diffusion coefficient of CO_2_ in MEA absorbent feed stream (m^2^ s^−1^)
$d_{h,i}$	Equivalent hydraulic diameter of channel (*m*), i=spiral, empty
E	Deviation of experimental results from the theoretical predictions
$E_{spiral}$	Further absorption flux enhancement
$f_{F}$	Fanning friction factor
h	Henry’s law constant (atm L gmol^−1^)
$H_{c}$	Dimensionless Henry’s constant
$H_{i}$	Hydraulic dissipate energy (J kg^−1^), i=spiral, empty
$I_{E}$	Absorption flux improvement
$I_{P}$	Power consumption relative index
$k_{a}$	Mass transfer coefficient in the CO_2_/N_2_ gas feed stream (m s^−1^)
$k_{b}$	Mass transfer coefficient in the MEA absorbent feed stream (m s^−1^)
$K_{ex}$	Equilibrium constant
$K_{ex}^{\prime }$	Reduced equilibrium constant
$K_{m}$	Overall mass transfer coefficient of membrane (m s^−1^)
${lw}_{f,i}$	Friction loss (J kg^−1^), $i=CO_{2}/N_{2},\;MEA$
L	Channel length (m)
$M_{w}$	Average molecular weight of CO_2_ and N_2_ gas mixture $({kg\;mol}^{-1}$)
$N_{exp}$	Number of experimental measurements
$P_{1}$	Saturation vapor pressure in the gas feed flow side (Pa)
$P_{2}$	Saturation vapor pressure in the liquid absorbent flow side (Pa)
$q_{a}$	Volumetric flow rate of the gas feed stream ($m^{3}s^{-1}$)
$q_{b}$	Volumetric flow rate of the MEA absorbent stream ($m^{3}s^{-1}$)
R	Gas constant (8.314 $J\;{mol}^{-1}K^{-1}$)
Re	Reynolds number
$r_{ai}$	Inside radius of inner tube (m)
$r_{ao}$	Outside radius of inner tube (m)
$r_{bi}$	Inside radius of shell tube (m)
$r_{p}$	Membrane pore radius (m)
Sc	Dimensionless Schmidt number
${Sh}^{s}$	Enhanced dimensionless Sherwood number
${Sh}_{lam}$	Dimensionless Sherwood number for laminar flow
$W_{p}$	Spiral ring pitch width (m)
$W_{r}$	Ratio of the various pitches inserted
J	Absorption flux ($mol\;m^{-2}s^{-1}$)
${\left|Y_{m}\right|}_{ln}$	Natural log mean CO_2_ mole fraction in the membrane
z	Axial coordinate along the flow direction (m)
*Greek letters*	
$\alpha^{s}$	Mass transfer enhancement factor
$\delta_{m}$	Thickness of membrane (µm)
ε	Membrane porosity
$\bar{v}$	Average velocity (${ms}^{-1}$)
$\rho_{i}$	Density (${kg\;m}^{-3}$), $i=CO_{2}/N_{2},\;MEA$
$\gamma_{m}$	Concentration polarization coefficients
*Subscripts*	
1	Membrane surface in CO_2_/N_2_ gas feed side
2(l)	Liquid phase on membrane surface on MEA feed stream
2(g)	Gas phase on membrane surface on MEA side
*a*	CO_2_/N_2_ gas feed stream
*b*	MEA absorbent feed stream
*cor*	Correlated results
*spiral*	Inserting spiral rings as promoters
*empty*	Empty channel
*exp*	Experimental results
g	Gas feed stream
*in*	At the inlet
l	MEA absorbent feed stream
*out*	At the outlet
*theo*	Theoretical predictions
*Superscripts*	
*as*	Ascending spiral ring pitch operations
*con*	Constant spiral ring pitch operations
*des*	Descending spiral ring pitch operations
